# Anti-Quorum Sensing Activity of Substances Isolated from Wild Berry Associated Bacteria

**Published:** 2017

**Authors:** Suha M. Abudoleh, Adel M. Mahasneh

**Affiliations:** 1. Faculty of Pharmacy, Isra University, Amman, Jordan; 2. Department of Biological Sciences, Faculty of Science, The University of Jordan, Amman, Jordan

**Keywords:** Quorum Sensing, *Chromobacterium violaceum*

## Abstract

**Background:**

Quorum Sensing (QS) is a mechanism used by bacteria to determine their physiological activities and coordinate gene expression based on cell to cell signaling. Many bacterial physiological functions are under the regulation of quorum sensing such as virulence, luminescence, motility, sporulation and biofilm formation. The aim of the present study was to isolate and characterize Quorum Sensing Inhibitory (QSI) substances from epiphytic bacteria residing on wild berries surfaces.

**Methods:**

Fifty nine bacterial isolates out of 600 screened bacteria were successfully isolated. These bacteria were obtained from berry surfaces of different plants in the wild forests of Ajloun-Jordan. Screening for QSI activity using *Chromobacterium violaceum* ATCC 12472 monitor strain, resulted in isolating 6 isolates exhibiting QSI activity only, 11 isolates with QSI and antibacterial activity, and 42 isolates with antibacterial activity only. Three potential isolates S 130, S 153, and S 664, were gram positive rods and spore formers, catalase positive and oxidase negative. These were chosen for further testing and characterization.

**Results:**

Different solvent extraction of the QSI substances based on polarity indicated that the activity of S 130 was in the butanol extract, S 153 activity in both chloroform and butanol; and for S 664, the activity was detected in the hexane extract. The chloroform extract of S 153 and hexane extract of S 664 were proteinaceous in nature while QSI substances of the butanol extract of S 130 and S 153 were non-proteinaceous. All the tested QSI substances showed a marked thermal stability when subjected at several time intervals to 70°*C*, with the highest stability observed for the butanol extract of S 153. Assessing the QSI substances using violacein quantification assay revealed varying degrees of activity depending upon the extracting solvent, type of the producer bacteria and the concentration of the substances.

**Conclusion:**

This study highlighted the potential of untapped reservoirs in nature to be used as a source of unique metabolite that may be further developed for therapy. The potential QSI substances included in this study are just one aspect to be further analyzed for use as biopharmaceutical agents.

## Introduction

Quorum Sensing (QS) is a cell density mediated mechanism used by bacteria to synchronize behavior on a population wide scale. This includes physiological activities and gene expression coordination based on cell to cell signaling 1. Small diffusible molecules, generally called auto-inducers, play the major role in this signaling pathway 2. Mostly all auto-inducers, regardless of their chemical nature, act in the same manner 3. The bacteria produce auto-inducers which diffuse extracellularly and accumulate in the surroundings until they reach a certain threshold concentration, after which it diffuses back to the cell regulating the expression of different genes 4–6. QS gene expression is regulated in the entire bacterial community 7, and by this, the bacteria will act as a multicellular organism which have several advantages over individual bacterial cells 8,9. Many bacterial physiological functions are under the regulation of quorum sensing such as virulence 10, luminescence 11, motility 12, sporulation and biofilm formation 13.

The term “anti-pathogenic compounds” has attracted great interest in the medical field. It refers to those substances that do not kill the bacteria and do not lead to the development of resistant strains over time 13,14 like the way many bactericidal substances do. They act on attenuation of bacterial virulence, preventing the establishment of a successful infection 9,15. *Chromobacterium violaceum* (*C. violaceum*) is the most used bacterial species as a monitor strain for studying quorum sensing inhibition activity. This bacterium has the ability to produce a violet pigment as a result of quorum sensing 5,16. QS Inhibitors (QSIs) will lead to loss of the violet pigmentation without actually killing the bacterial cells. Therefore, it acts as a good QS Inhibition screening candidate 12.

The most widely known QSIs are the halogenated furanones which have many limitations for human use due to their toxic side effects and carcinogenic properties making them unsuitable for pharmaceutical usage 16,17. Accordingly, a lot of researches are looking into discovering new chemicals that have QSI activity with reduced or absent side effects 9,14,16,18.

The aim of this study was to search through nature’s own rich collection of chemicals in order to find new substances that act as anti-pathogenic drugs. Among sources of these potential new chemicals are the bacteria in different habitats especially unusual niches. The surfaces of berries found in natural forests hold large populations of bacteria that have evolved a variety of adaptations to exist in such continuously changing environments. Moreover, limited space and nutrient availability act as driving forces for the production of a variety of metabolites that reduce competition between bacterial communities on the surface of the same berry.

Recently, *Bacillus* of different origins is given special interest in the quest for antimicrobial and anti-pathogenic materials 19. Alasil *et al* stressed the importance of screening different spore-forming bacteria for such materials with special emphasis on *Bacillus* and *Paenibacillus* isolates. Since several virulence capabilities in bacteria are under QS-control, it became logical to search for anti-quorum sensing substances of different origins. In this report, culture extract of three spore-forming aerobic bacterial isolates from the surface of wild forest berries was tested for their QS inhibitory effects in a *C. violaceum* ATCC 12472 monitor strain. Production of QSI and partial characterization of the extracted crude QSI are presented and discussed.

## Materials and Methods

### Collection of wild forest berries

Wild berries were collected under aseptic conditions from Ajloun wild forests, in the period between August and September 2010. The samples were collected in sterile laboratory samplers, and kept in an ice box to conserve freshness until it reached the laboratory. The berries included *Piatacia palastina Boiss, Crataegus aronia (L.), Ceratonia siliqua L., Rosa phoenicia, Pyrus suriaca Boiss, Crataegus siniacia, arbutus andrachne, Rhamnus alaternus L. Rhamnus vlycioides L*. and *Schnus molle L*.

### Preparation of berries

Ten grams of each berry type were washed three times by sterile distilled water in order to remove loosely attached bacteria. To detach epiphytic bacteria, the washed berries were added to 20 *ml* of sterile normal saline with a few drops of Tween 20 and left in an orbital shaker over night.

### Bacterial isolation

Serial decimal dilutions of the detached bacteria were prepared in sterile saline. From each dilution, 100 *μl* were spread over nutrient agar plates in triplicates. The plates were then incubated at 30°*C* for 48–72 *hr*. Colonies of different shapes were picked and subcultured further onto nutrient agar plates. Three subcultures were employed to ensure purity of the single isolates, and further checked for spore-forming ability using Malachite green stain.

### Screening for anti-quorum sensing activity

For the QSI screening, spore forming epiphytic bacterial isolates were streaked onto Lauria-Bertani (LB) agar either in a straight line or circular streak and incubated for 24 *hr* at 30°*C* according to the method of Mc-Lean *et al* 20. The monitor strain *C. violaceum* (ATCC 12472) was grown separately in 5 *ml* of LB broth medium and incubated at 30°*C* overnight. The monitor strain (100 *μl*) from a culture (OD_600_=0.1) was prepared in a semisolid agar (0.75% agar) of 10 *ml* volume for an overlay on top of the selected target bacteria. These plates were incubated at 30°*C* for 24 *hr*. A positive QSI result was indicated by inhibiting violacein pigmentation of the monitor strain around the streak of the test bacteria. Negative results were indicated by the presence of pigmentation.

### Identification of active epiphytic bacteria

Active epiphytic bacterial isolates were partially identified using gram stain reaction, spore formation, catalase and oxidase production.

### Production of crude quorum sensing inhibitory substances

For each isolate, one colony was cultured in 5 *ml* LB broth for reactivation. One milliliter of the activated bacteria was then transferred into a 250 *ml* flask containing 100 *ml* of LB broth and was incubated in an orbital shaker at 30°*C* and 100 *rpm* for 48–72 *hr*. At the end of the incubation time, the 100 *ml* of bacterial cultures were centrifuged at 3800 *rpm* and for 30 *min*. The resulting supernatant was filtered using sterile syringe filter (0.45 *μm* pore size) for further analysis 21.

### Testing quorum sensing inhibition activity in the supernatant

The activity of the sterile supernatant was tested using agar well diffusion method according to the method of McLean *et al* 22 and Lamberte *et al* 23; in brief, LB agar plates were inoculated with 1 *ml* of *C. violaceum* (OD_600_= 0.1) in order to form a lawn of bacteria. The plates were kept refrigerated for one hour to allow the absorption of the broth. Sterile Cork borer (8 *mm* diameter) was used to puncture the plates making multi-welled plates. To each well, 200 *μl* of each supernatant were introduced aseptically and the control well contained sterile distilled water. The plates were then incubated at 30°*C* for 24 *hr*. The clear halo free of bacterial growth around the well indicated growth inhibition while turbid halo (disappearance of violet color only) indicated QSI activity.

### Extraction of QSI substances

Bacterial supernatants of S 130, S 153, and S 664 were extracted separately using organic solvents of increasing polarity (chloroform, hexane and 2-butanol) using the following procedure: bacterial supernatant was first mixed with chloroform using a 1:1 ratio in a separatory funnel and then the solvent and supernatant were allowed to separate. Chloroform phase was removed from the funnel into a separate flask. The process was repeated using hexane followed by 2-butanol on the same supernatant. Each organic solvent was then evaporated under vacuum and the concentrate was stocked for further analysis and testing 24,25.

### Proteinase activity

Proteinase K was used to test its effect upon the QSI substances according to Nithya *et al* and Wilson *et al*. The extracts were incubated with 1 *mg/ml* of proteinase K for 18 *hr*. The activity of the extracts was then tested using well diffusion method as described by Lamberte *et al*.

### Temperature stability

Heat stability of the crude QSI substances was tested at 70°*C* at different exposure time intervals (0, 5, 10, 15 and 30 *min*) according to Nithya *et al* after which the activity of the heated extracts was tested using well diffusion method.

### Violacein production

***Flask incubation assay:*** A culture broth of *C. violaceum* ATCC 12472 with an OD of 0.1+0.02 was prepared in LB broth. One *ml* from this broth was inoculated into each of 6 separate Erlenmeyer flasks each containing 18 *ml* LB broth and supplemented with 1 *ml* of different QSI extracts at concentrations of each QSI extract (100, 75, 50, 37.5, 25 and 12.5 *mg/ml*). For the control flask, 1 *ml* of sterile distilled water was added. The flasks were incubated in an orbital shaker at 30°*C* and 150 *rpm* for 18 *hr* for violacein quantification 5,16.

***Quantification of violacein production:*** From each flask, 1 *ml* was centrifuged at 13000 *rpm* for 10 *min* to precipitate insoluble violacein. The supernatant was discarded and 1 *ml* of DMSO was added to the pellet, vortexed vigorously until the violacein was completely solubilized, then centrifuged again at 13000 *rpm* for 10 *min* to remove the cells. The absorbance was read at 585 *nm*, and the treated groups were compared with the control group 16. The inhibition percentage was calculated as follows:

$$\frac{\text{Inhibition}\%={\text{OD}}_{585}\;\text{of}\;\text{control}-{\text{OD}}_{585\;}\text{of}\;\text{treated}\;*100\%}{{\text{OD}}_{585}\;\text{of}\;\text{control}}$$

***Confirmation of the quorum sensing inhibitory activity:*** After 24 *hr* of incubation in the flask assay, 100 *μl* from each flask were spread on nutrient agar after being diluted to confirm the anti-quorum sensing activity and exclude any antibacterial activity. The plates were incubated at 30°*C* for 24 *hr* and bacterial counts were compared with control group.

### Statistical analysis

The results were presented as mean±SD of three independent experiments. Statistical differences were determined by one way ANOVA followed by Dunett’s test and unpaired t-test using Graph pad Prism software. Differences were considered significant at p≤0.05.

## Results

### Bacterial isolation

From several screening runs, 600 distinct colonies that varied in size, pigmentation, texture, elevation, and margin surface originating from wild berries surfaces were picked to be screened further for anti-quorum sensing activity.

### Screening for anti-quorum sensing activity

**Figure 1.**
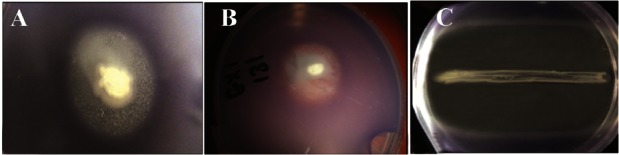
A and B QSI activity of epiphytic bacteria isolates S130 and S131, the QSI activity is indicated by loss of the violet color of *C. violaceum* ATCC 12472. In C: both growth inhibition and QSI activity of isolate S 396.

All 600 isolates were screened to choose those with potential anti-quorum sensing activity. Out of 600 isolates, 59 isolates with both antibacterial and QSI activity were successfully isolated. Six isolates out of 59 exhibited QSI activity, without any effect on bacterial growth. Eleven isolates had both QSI and bacterial growth inhibition activity against the monitor strain *C. violaceum* ATCC 12472. Fourty two isolates exhibited growth inhibitory activity. Figure 1 shows examples of the activity of 3 out of the 59 isolates that showed antibacterial and QSI activity.

### Partial identification of potential isolates

In the isolates 59 were gram positive with 93% gram positive rods and 7% were gram positive spherical bacteria. The spore formation ability was detected in 80% of the isolated epiphytic bacteria. Most of the isolates (95%) were catalase positive and 7% of these isolates were oxidase positive.

### Productions of crude quorum sensing inhibitory substances

**Figure 2.**
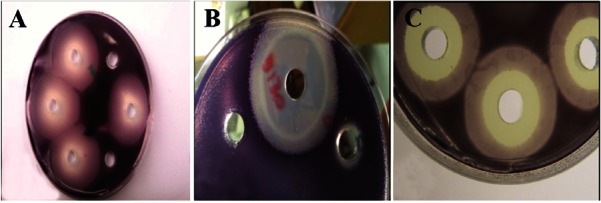
The QSI activity of different epiphytic bacteria, the activity was tested in the supernatant of the potential isolates. A: only QSI activity, B and C: QSI and growth inhibition activity.

Testing the production of the active substances in the supernatant of the producer strains was carried out. Three aerobic spore forming isolates that showed the best results were chosen and coded S130, S153, and S 664. These isolates were tested for the optimal production of the QSI substances which reached maximum activity after 48 h for isolate S 130 and S 153 and 72 *hr* for isolate S 664 (Figure 2).

### Extraction of QSI substances

The activity of the prepared extract was tested to determine which of the organic solvents yielded active ingredients (Table 1). Isolate S 153 exhibited QSI activity in both chloroform and butanol extracts. However, higher yields of butanol extract are recorded. As for the other isolates, S 130 and S 664, the QSI activity was only detected with butanol and hexane, respectively and both yields were relatively high (Table 1).

**Table 1.** Crude extracts activity and yields of different isolates

**Crude extract activity and yield ( * g/L * )**			

** Isolate **	** Hexane **	** Chloroform **	** Butanol **
** S 130 **	0.0	0.0	2.5
** S 153 **	0.0	0.2	2.0
** S 664 **	1.0	0.0	0.0

### Proteinase activity

In order to determine whether the active fractions of the different isolates were proteins or not, the extract was subjected to proteinase k and incubated at 37°*C* for 18 *hr*, after which the activity of the extracts was tested using agar well diffusion method the activity was expressed as diameter of the hydrolysis zone (Table 2). The results indicate that the nature of chloroform extract of S 153 and hexane of S 664 was proteinaceous, while butanol extract of both S 153 and S 130 was of non-proteinaceous nature.

**Table 2.** Effect of proteinase K on the crude QSI substances. Results were expressed as mean±SD

	**Diameter of inhibition zone ( * mm * )**			

	** Chloroform S 153 **	** Butanol S 153 **	** Butanol S 130 **	** Hexane S 664 **
** With proteinase K **	-	23±2.9	21r1.0	-
** Without proteinase K **	20±1.2	28±0.6	25r1.0	29±0.6

No activity.

### Temperature stability

**Figure 3.**
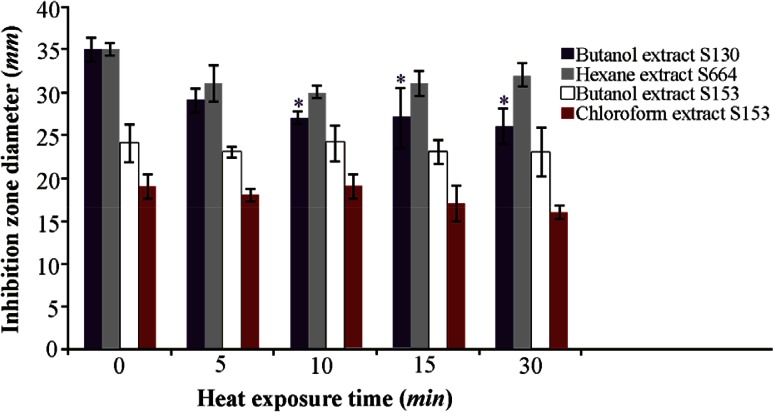
Thermal stability of the QSI substances of different isolates at 70°*C* after different exposure times. Results are expressed as mean±SD, Significant result * at p≤0.05.

To test the thermal stability of the active extract, it was subjected to 70°*C* for different durations of time. The butanolic extract of S 153 retained 100% activity after 30 *min* exposure at 70°*C*, while 74, 91 and 84% activity were retained for the butanolic extract of S130, hexane extract of S 664 and chloroform extract of S 153, respectively (Figure 3).

### Quantification of violacein production

**Figure 4.**
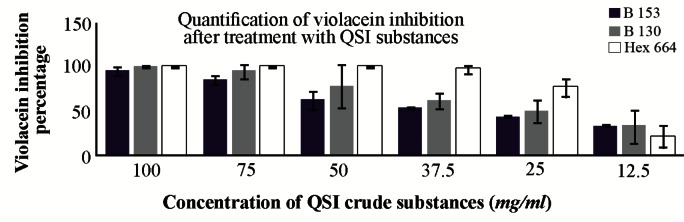
Inhibition percentage of violacein exerted by bacterial QSI substances at different concentrations. B130: butanolic extract of isolate S 130, B153: butanolic extract of isolate S 153, hex 664: hexane extract of isolate S 664

**Figure 5.**
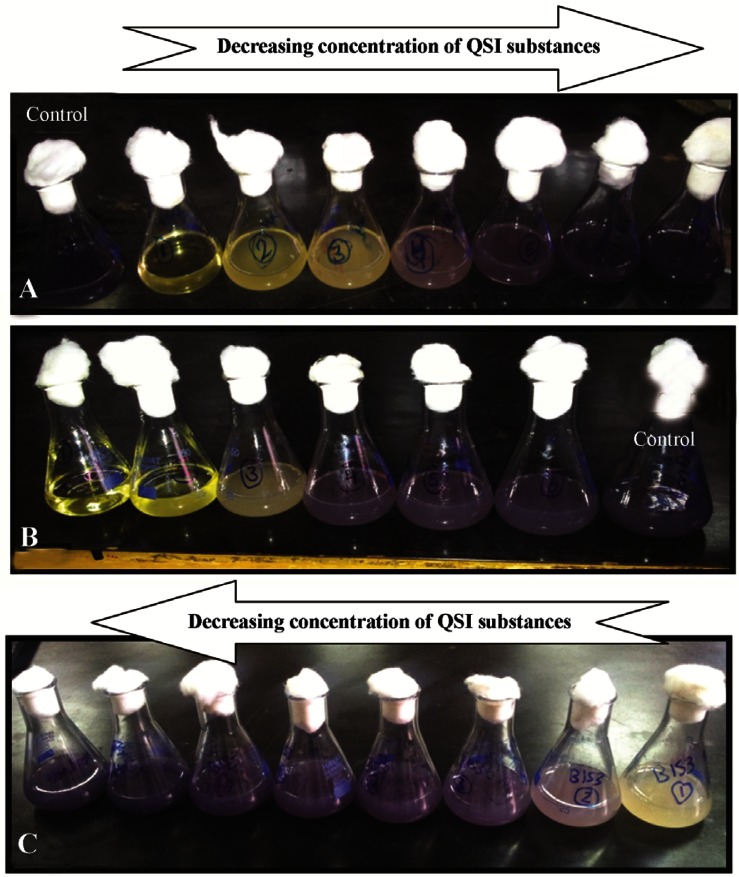
Quantification of violacein by flask method, A: hexane QSI substances of isolate S 664 extract, B: butanolic QSI substances of isolate S 130 extract, and C: the QSI substances of isolate S 153 butanolic extract.

The results of the inhibition percentage of violacein production are summarized in a bar graph in figure 4. Figure 5 shows the flask method assay that was used for the quantification of violacein.

### Confirmation of the quorum sensing inhibitory activity

**Figure 6.**
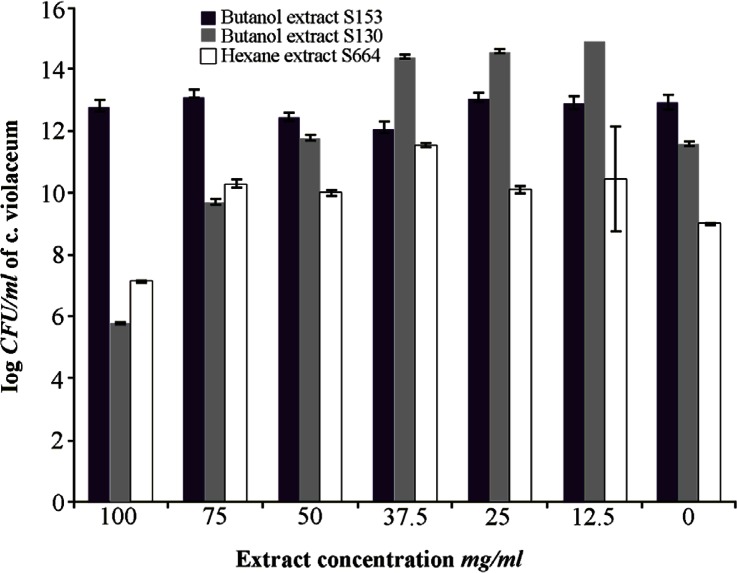
Effects of QSI substances on bacterial growth expressed as log CFU of *C. violaceum* ATCC 12472 violacein inhibition assay. The results are expressed as mean±SD *significantly lower than control, ** significantly higher than control, p≤0.05.

The results indicated concentration- dependent inhibitory activity in all tested extracts (Figure 3). Bacterial count performed on LB agar plates incubated at 30°*C* for 24 *hr* showed no significant difference (p≤0.05) in the number of colony forming units in most tested concentrations except that for the higher concentrations of the butanol extract of S 130 and the hexane extract of S 664. The results are expressed as log CFU and compared with control untreated group (Figure 6). These results substantiate the anti-quorum sensing activity of the extract of bacterial isolates rather than any other effects such as bacterial growth inhibition.

## Discussion

The therapeutic efficacy of antibiotics correlates closely with their bactericidal effect 26. Unfortunately, bacteria can adapt to the selective pressure from antibiotics *via* genetic alteration leading to development of antibiotic resistance 27. The efficiency of many traditional antibiotic treatments is currently decreasing, and the occurrence of multiple antibiotic-resistant pathogenic bacteria is increasing in an uncontrollable rate 28. Attenuation of bacterial virulence and the use of anti-pathogenic materials rather than killing the pathogen has become a new concept for control of bacterial infections. This mode of action and the use of such materials would not impose a selective pressure for the development of bacterial resistance to the antibiotic 27, and would open new research areas in the search for new therapeutic drugs.

One of the main aims of this type of research is to find novel unexploited natural areas to be the source for potential materials as probable producers of new drugs. Natural forests are a good choice for such isolates. The habitats in such areas are still a good source of novel microbial isolates with far-reaching drug potential.

Plant surfaces hold a huge diverse number of microorganisms that are restricted to a relatively small surface area 29. These microorganisms have developed certain adaptations in order to cope with the surrounding conditions of varying environmental and other conditions to ensure their survival 30. As a consequence, they have adapted to produce metabolites that would be used in an array of applications including new pharmaceuticals which may be of critical importance in the quest for new drugs.

The screening protocol followed in this study resulted in isolating several bacterial isolates 59 that were capable of producing antibacterial materials 42, or anti-pathogenic substances 6, and eleven isolates had both QSI and bacterial growth inhibitory activity against *C. violaceum* ATCC 12472. Isolates S 130, S 153, and S 664 were selected for further testing for their ability to produce anti-quorum sensing materials. Tentative identification indicated these to be aerobic gram positive spore-forming bacteria.

Weng *et al* 31 used the soil from land and beaches as a reservoir of isolating microorganisms. Out of their 500 isolated bacterial strains, 12 potentially QSI-active isolates were successfully isolated. Alvarez *et al* 13 found that the tea tree and rosemary extract exhibited both antibacterial and QSI activity. Kanagasabhapathy *et al* 21 isolated 96 epiphytic bacteria from marine algae. Twelve percent of the isolated bacteria were able to produce QSI. Abed *et al* 32 isolated QSI compounds from extremophilic microorganisms. In addition, Ma *et al* 33 isolated 1177 tobacco leaf-associated bacteria; 14% of the isolates exhibited QSI activity by interfering with Acyl Homoserine Lactone (AHL) activity which is an autoinducer. One hundred and six isolates inhibited QS by enzymatic degradation of AHL, while the remaining isolates inhibited QS by other chemical ways of inhibition. The QSI activity that was observed against *C. violaceum* ATCC 12472 by the tested extracts was mainly due to inhibition of the QS mechanism either by antagonistic activity of AHL or by destruction of the AHL 33.

Studying the productivity of the tested isolates (S 130, S 153 and S 664) indicated maximum production of QSI substances in culture media at 48–72 *hr*. Similar results were reported by Thenmozhi *et al* 34 where the incubation period required for QSI production by the bacteria was 48 *hr*, while 72 *hr* period was needed to reach the optimum productivity of marine-isolated bacteria by Monsson *et al* 35.

Subjecting QSI substances to polar extraction with different organic solvents including hexane, chloroform, and butanol, resulted in pooling QSI substances that shared characteristics of similar compounds or substances. Nithya *et al* 10 isolated QSI substances from bacteria originating from different environments. These QSI substances with respect to their origin are indicative of response of such bacteria to harsh environments as in the case with wild plant surfaces 29,30. Adonizio *et al* 36 studied the activity of different South Florida plants against AHL production by *C. violaceum*. The unwashed plant leaves (containing epiphytic bacteria) showed higher QSI activity than their washed counterparts which indicated enhancement of QSI activity by epiphytic bacteria. Al-Hussaini *et al* 37 isolated QSI substances from *Laurus nobilis* with the butanol fraction having prominent activity.

Results of polarity extraction of S 153 QSI substances were in accordance with the result of Wilson *et al* 25 where the tested anti-microbial activity (both antimicrobial and anti-quorum sensing) was found in multiple organic phases. These results indicated the presence of QSI substances with different polarities 25. The yields of these extracts varied depending upon the isolate type and the extraction solvent: the butanol extract gave the highest yield (2.5 and 2.0 *g/L*) for both isolates S 130 and S 153, respectively; the chloroform extract showed the lowest yield (0.2 *g/L*), and hexane extract yields were in between (1.0 *g/L*).

Characterization of the QSI substances is a necessary step in substantiating the use of such materials as anti-pathogenic tools. As a result, proteinase K was used to determine the nature of the active material, whether proteinaceous or otherwise. It was clear that the nature of the QSI substances varied in this case; some bacterial isolates of QSI were of proteinaceous nature as in the case of the chloroform extract of S 153 and the QSI substances of hexane extract of S 664. Mustafa *et al* 5 found that the nature of QSI materials of tested *Bacillus* spp. were non proteinaceous. Nithya *et al* 10 found that the QSI substances released by *Bacillus pumilus* were protein in nature, which agrees with findings of this study on spore forming gram positive rods.

Testing thermal stability of these QSI substances indicated that the substance of the butanol extract of S 153 was the most stable substance among the others and it retained almost full activity after being subjected to 70°*C* for 30 *min*. Moreover, the QSI substances of chloroform extract of S 153 showed no significant changes after being subjected to 70°*C* for 30 *min*. The varying thermal tolerance and stability of the QSI substances observed in this study agree in part with the findings of Mustafa *et al* 5 where the QSI extract activity was fully retained after 15 *min* exposure to 90°*C*. On the other hand, the QSI substances of *Bacillus pumilus* lost their activity after the same thermal treatment. Vogt *et al* 38,39 studied the thermal stability of different proteins, and they reported that increasing hydrogen bonding in proteins is considered the most important factor for thermal stability, a factor that is not tackled in this study.

In quantitative analysis of the selected QSI substances against violacein production by *C. violaceum* ATCC 12472, the butanol extract of S 153 reduced the violacein production dramatically to the level of 95% at the higher concentration (100 *mg/ml*), and the lowest inhibitory percentage at concentration of 12.5 *mg/ml* was 32.5%. These violacein inhibitory percentages were due to QS blockage mechanism rather than killing the bacteria which was confirmed by counting the CFU of the *C. violaceum* ATCC 12472 at that concentration. On the other hand, when *C. violaceum* ATCC 12472 was treated with different concentrations of butanol extract of S 130, the violacein inhibition percentage was 100 and 95% at concentrations of 100 and 75 *mg/ml* of the QSI substances, respectively. These violacein inhibitory percentages were due to killing of the bacteria rather than inhibiting the QS mechanism and this is attributed to significant reduction in the CFU at these two concentrations. Taganna *et al* 40 found that at certain extract concentrations of *Terminalia catappa,* the extract significantly increased the growth of *C. violaceum* with significant reduction in the violacein production, which is reduced due to QS blockage. This result correlates well with the study of Choo *et al* 16 where they found that vanilla extract reduced the violacein production up to 98%, and the result of Packiavathy *et al* 12 where the *Cuminum cyminum* exhibited 90% inhibition of violacein production. Vattem *et al* 41 found that the extract of *Rosmarinus officinalis* leaves inhibited the violacein production by 40%. Rasmussen *et al* 42 found that the methanolic extract of *Medicago truncatula* exhibited QSI activity. The results of Choo *et al’s* 16 study with respect to violacein inhibition agree with our findings and similar results were obtained by Thenmozhi *et al* 34.

These results definitely indicated the possibility of isolation of QSI substances from natural environments including microflora associated with different parts of the plants including the berries. QS inhibition is an alternative route for pathogen control through manipulation of gene expression rather than killing the pathogen. Use of QSI substances will no doubt enhance the fight against emerging resistant pathogenic bacteria. The field is still at the beginnings and further screening programs and testing protocols are needed.

## Conclusion

The results of this study revealed that the components of nature are still the untapped reservoir for a wide variety of medicinal applications. It also emphasizes the role of epiphytic bacteria as being the potential source for unique metabolites that are needed for alternative therapies. The QSI substances are just one aspect to be further investigated and developed in the everlasting quest for new chemotherapeutic agents.
